# Diabetes Mellitus and Risk of Thyroid Cancer: A Meta-Analysis

**DOI:** 10.1371/journal.pone.0098135

**Published:** 2014-06-13

**Authors:** Yohwan Yeo, Seung-Hyun Ma, Yunji Hwang, Pamela L. Horn-Ross, Ann Hsing, Kyu-Eun Lee, Young Joo Park, Do-Joon Park, Keun-Young Yoo, Sue K. Park

**Affiliations:** 1 Department of Preventive Medicine, Seoul National University College of Medicine, Seoul, Korea; 2 Cancer Research Institute, Seoul National University, Seoul, Korea; 3 Department of Biomedical Science, Seoul National University Graduate School, Seoul, Korea; 4 Cancer Prevention Institute of California, Fremont, California, United States of America; 5 Department of Internal Medicine, Seoul National University College of Medicine, Seoul, Korea; 6 Department of Surgery, Seoul National University College of Medicine, Seoul, Korea; University of Bari Aldo Moro, Italy

## Abstract

**Introduction:**

Diabetes mellitus (DM) is an important risk factor for endocrine cancers; however, the association with thyroid cancer is not clear. We performed a systematic review and meta-analysis to clarify the association between thyroid cancer and DM.

**Methods:**

We searched MEDLINE, PUBMED and EMBASE databases through July 2012, using search terms related to diabetes mellitus, cancer, and thyroid cancer. We conducted a meta-analysis of the risk of incidence of thyroid cancer from pre-existing diabetes. Of 2,123 titles initially identified, sixteen articles met our inclusion criteria. An additional article was identified from a bibliography. Totally, 14 cohort and 3 case-control studies were selected for the meta-analysis. The risks were estimated using random-effects model and sensitivity test for the studies which reported risk estimates and used different definition of DM.

**Results:**

Compared with individuals without DM, the patients with DM were at 1.34-fold higher risk for thyroid cancer (95% CI 1.11–1.63). However, there was heterogeneity in the results (p<0.0001). Sensitivity tests and studies judged to be high quality did not show heterogeneity and DM was associated with higher risk for thyroid cancer in these sub-analyses (both of RRs = 1.18, 95% CIs 1.08–1.28). DM was associated with a 1.38-fold increased risk of thyroid cancer in women (95% CI 1.13–1.67) after sensitivity test. Risk of thyroid cancer in men did not remain significant (RR 1.11, 95% CI 0.80–1.53).

**Conclusions:**

Compared with their non-diabetic counterparts, women with pre-existing DM have an increased risk of thyroid cancer.

## Introduction

Thyroid cancer incidence has been increasing worldwide since the early 1980s, most dramatically in the past decades [1], [2]. In Korea, the incidence rate of thyroid cancer in adults was about 91 per 100,000 persons in 2010, substantially higher than anywhere else in the world. Thyroid cancer has been most common cancer occurring in Korea since 2005, especially among women. In 2009, a total of 31,811 new incident thyroid cancers were diagnosed; 73.9% (26,682 cases) in women (2009 Cancer Registry data from Korea National Cancer Center). Despite the increase in incidence, the thyroid cancer survival remains high [3], [4].

Risk factors for thyroid cancer are not well established. Only neck irradiation and for follicular thyroid cancer, insufficient iodine intake, are known risk factors for thyroid cancer [5]–[7]. The dramatically increasing incidence of thyroid cancer might be partly attributed to detection bias due to increasing screening by neck ultrasound; however the increase cannot be fully explained by increased medical surveillance or improved detection methods alone [8]. The role of other risk factors in the development of thyroid cancer and in its increasing incidence needs further elucidation. Here we address the possible role of diabetes mellitus (DM).

Type II DM is one of the most rapidly increasing public health issues in Korea, as well as elsewhere. The prevalence of DM is expected to an increase from 2.8% in 2000 to 4.4% in 2030, with the rate increase being greater in developing countries than in developed ones [9]. In Korea, the prevalence rate of DM in adults 30 years of age and older has increased from 1–4% in the 1970s to 9.5% in 2007 (the National Nutrition Survey) [10]. DM has been associated with an increased risk of several types of cancer, including pancreas [11], liver [12], and endometrium. While several observational studies have previously examined cancer risk or mortality [13]–[16], the results in relation to thyroid cancer have not been consistent, due largely to the small number of incidence cases of thyroid cancer in any given study [13], [17], [18]. Although two recent large-sized population-based longitudinal studies indicate that a history of DM may be a risk factor for thyroid cancer [19], [20], this risk may be overestimated in the study of Radiologic Technologists as this is a high risk population. Thus, the present review and meta-analysis was designed to determine whether type II DM effects thyroid cancer incidence and whether the effects differ by gender.

## Materials and Methods

### Search strategy

We performed a literature search up through August 2012 using the PubMed, Medline and EMBASE databases with the following search terms: (diabetes and thyroid cancer), (diabetes and cancer) (diabetes and thyroid), (type 2 diabetes and cancer), (thyroid cancer and fasting glucose), (thyroid cancer and hyperglycemia), (thyroid cancer and risk factor) and (thyroid cancer and metabolic syndrome). Furthermore, to find any additional published studies, a manual search was also performed by checking all the references of all the studies. All studies included in the meta-analysis were scored for quality using the quality reporting standards for meta-analyses outlined by Newcastle-Ottawa scale (NOS) [21].

### Literature search

**Figure 1:**
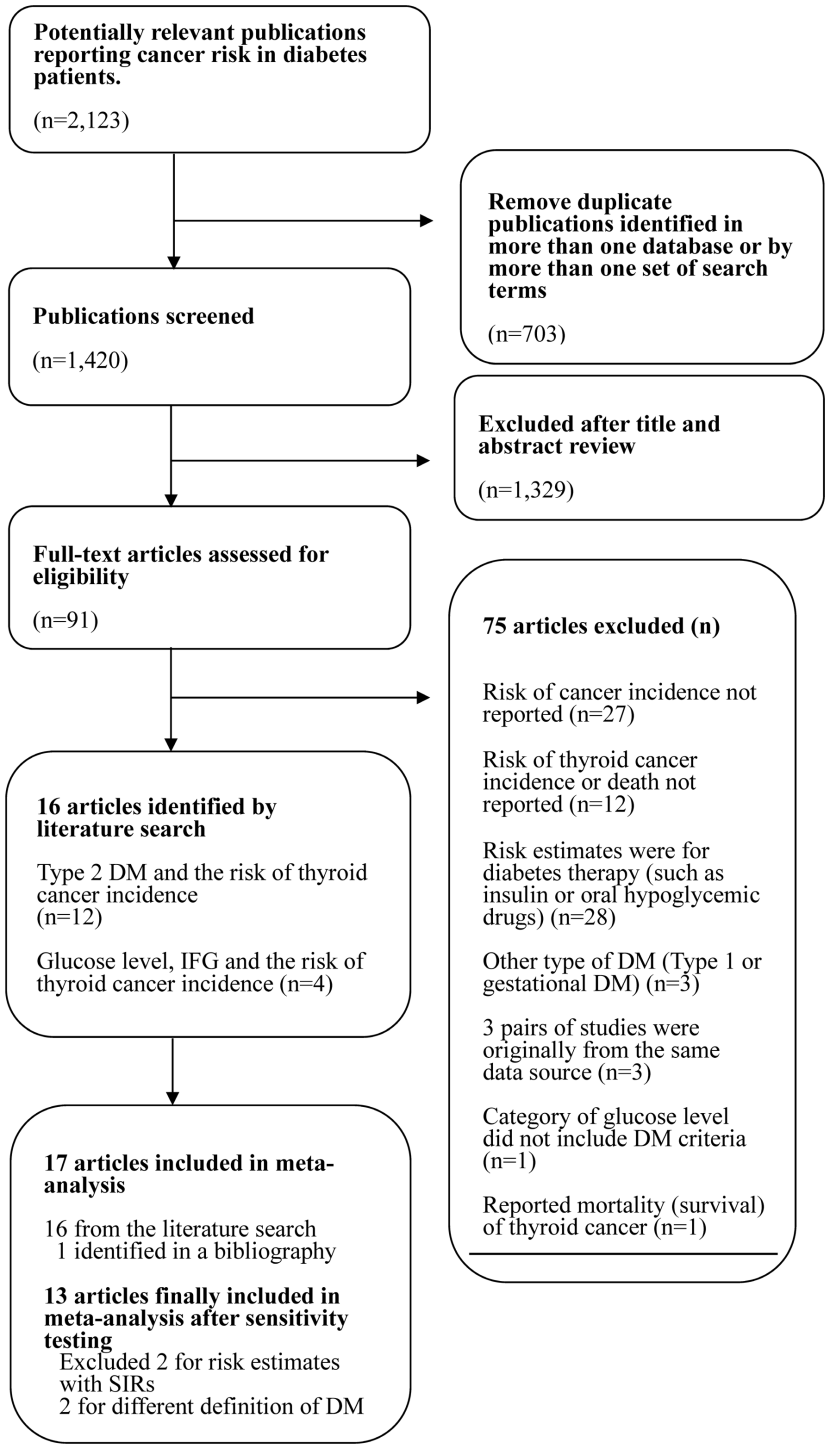
Literature search algorithm.

Of 2,123 articles originally identified, we excluded 703 duplicates (i.e., those that appeared in more than one database or from more than one set of search terms) (Figure 1). Another 1,329 articles were excluded after screening the title and abstract. For the remaining 91 articles, we conducted a full-text assessment for relevance. Of these 91, 75 studies were excluded as follows: the studies did not reported the risk of thyroid cancer incidence with a 95% CI (27 studies); the studies analyzed the effects of diabetes therapy (such as insulin or metformin) only (28 studies); the studies did report the risk of thyroid cancer incidence (12 studies); the studies did not address type II DM (3 studies) [22]-[24]; the study populations were originally from the same data source among 6 studies (3 studies) [25]–[27]; or the study examined only thyroid cancer mortality (1 study) [28]; or the categorical levels of glucose such as 2.2–4.1, 4.2–5.2, 5.3–6.0, 6.1–6.9, and 7.0 mmol/L+ (1 study) [29]. Of remained 16 relevant articles, one study [30] showed the results from five prospective studies such as NIH-AARP, USRT, PLCO, AHS, and BCDDP, however we included only PLCO study results in our meta-analysis because the source population from NIH-AARP and USRT were duplicated with the selected two studies [19], [20] and the risk estimates were not estimated from AHS and BCDDP due to few cases. Moreover, we found the additional article [31] by a manual search using the reference lists of 16 articles. Therefore, 17 studies were included in the meta-analysis.

### Study selection and assessment

Studies were required to meet the following inclusion criteria to be eligible for inclusion in the meta-analysis: case-control studies that recruited thyroid cancer cases and controls without thyroid cancer; or cohort studies conducted among healthy individuals or that were reconstructed among type 2 diabetic patients to estimate the thyroid cancer risk compared with the total population of the country where the study was performed. In addition, studies that compared type 2 DM patients to the source population in order to estimate the risk of thyroid cancer using the standardized incidence ratio (SIR) were also included but these studies were excluded [14], [32] when we performed sensitivity analysis.

The exposure of interest was the presence of pre-existing type 2 DM. If there was lack of the information about whether a diagnosis of type 2 DM had been made, we used the number of patients with impaired fasting glucose levels relative to the reference level identified by the WHO (100<fasting glucose<126 mg/dl). Crude risk estimates for the patients with impaired glucose metabolism, compared with normal glucose levels, were calculated. However, we excluded the studies which used a different or definition of DM [33] or an unavailable glucose level (quintile) in sensitivity analysis [34]. We used the mean level of glucose for each category to justify the patients in normal or abnormal glucose status. Furthermore, if the number of each category divided as the quartile of glucose level, we used the 95 confidence interval to infer the risk in one study [35]. The risk was recalculated by each level of 0.01 mmol/L for glucose level and we estimated the risk by summarized relative risks whose level were higher than the lower limit of confidence interval whom the glucose level were under the confidence interval as the reference.

The main outcome of interest was the reported odd ratios (OR), relative risk (RR), or hazard ratio (HR) or estimates and their corresponding 95% confident intervals (95% CIs). If risk estimates were only given for males and females separately, we recalculated the risk for all patients combined. There were 3 studies that provided risk estimates only for females [13], [35], [36] In addition, there were no observed incident cases among men in 2 studies [20], [35].

The following data were abstracted from each article: the first author's last name, publication year, country where the study was performed, study period, participant's age range, sample size (cases and controls or cohort size, and number with a past history of type 2 DM), variables adjusted for in the analysis, and the RRs and their 95% CIs. The countries with low or high incidence rates of thyroid cancer were classified according to Globocan comparing to the worldwide average incidence (4.0 age-standardized rate per 100,000) [37]. The countries with high incidence included United States [19], [20], [30], [36], [38], Iceland [35], Italy [18], Canada [39], Israel [40], Taiwan [41] and Turkey [33]. Other countries including Sweden [17], [32], [34], Norway [34], Denmark [14], and Japan [13], [31], were classified into “around or lower than worldwide average”. The quality of the study was assessed using the 9-star Newcastle-Ottawa Scale (range: 0 to 9) [21]. Data extraction was conducted independently by two investigators, with disagreements resolved by consensus.

### Statistical analysis

We used a random-effect model to obtain the summary relative risk and 95% CI for the association between DM and thyroid cancer risk. Statistical heterogeneity among studies (i.e., whether the differences obtained between studies was due to chance) was evaluated by using the Cochran Q and I^2^ statistics. For the Q statistic, a p-value<0.10 was considered statistically significant for heterogeneity; for I2, a value>50% is considered a measure of heterogeneity. All HRs from cohort studies and ORs from case-control studies were estimated as RRs. Publication bias was evaluated with the use of the Egger regression asymmetry test in which a p-value less than 0.05 was considered representative of statistically significant publication bias based on a funnel plot.

Subgroup analyses were performed according to the following characteristics: gender (males, females, combined); study designs (cohort or case-control); quality of the study methodology across the studies (6 or more, less than 6); and geographical area (high incidence of thyroid cancer, low incidence of thyroid cancer relative to the global average). All the sub-analyses were performed after excluding 4 studies using the risk estimates with SIRs [14], [32] and the different definition of diabetes [33], [34] for sensitivity analysis. All statistical analyses were performed with the STATA software, version 10 (Stata Corp, College Station, Texas).

## Results

Table 1 summarizes the study characteristics of the 17 studies included in the meta-analysis. Two studies [14], [32] out of 17 studies used SIRs as the measure of relative risk, and the other two studies [33], [34] used different definitions for DM (also included IFG and IGT or used quintile of glucose level). The 13 studies,which remained after sensitivity analysis, finally consisted of 2 case-control [18], [31] and 11 cohort studies [13], [17], [19], [20], [30], [35], [36], [38]–[41], published between 1992 and 2012. Six studies were performed in the United States and Canada [19], [20], [30], [36], [38], [39], three were in Europe (Sweden, Iceland and Italy) [17], [18], [35] and other four were in Asia (Japan, Israel and Taiwan) [13], [31], [40], [41]. Ten of the studies were deemed of high quality [13], [17], [19], [20], [30], [31], [35], [38], [39], [41] (Table S2).

**Table 1:** Details of studies on type 2 diabetes for thyroid cancer risk.

Author [Reference]	Design	Control type or reference population	Country	Age range	Study period	N of thyroid cancer cases	N of controls (or person years)	Comment
Cohort studies								
Aschebrook-Kilfoy et al. 2011 [19] NIH-AARP	Cohort	-	US	50–71	1995–2006	585		The NIH-AARP Diet and Health Study cohort [Study qualitya by (Selection:3, Comparability:1, Outcome:2)]
Wideroff et al. 1994 [14] b	Cohort	Danish population	Denmark	-	1977–1989	31	N/A	Standardized incidence rate Using Danish Central Hospital Discharge Register [Study qualitya by (Selection:3, Comparability:0, Outcome:2)]
Adami et al. 1991 [17]	Cohort	Swedish population	Sweden	-	1984–1991	19	N/A	Using the national population register. *[Study quality* a *by (Selection:4, Comparability:1, Outcome:2)]*
Chodick et al. 2010 [40]	Cohort	-	Israel	>21	2000–2008	114	(671,089)	Using MHS national registry of DM *[Study quality* a *by (Selection:4, Comparability:0, Outcome:1)]*
Inoue et al. 2010 [13]	Cohort	-	Israel	40–69	1990–2003	103 (Women)	(1,002,037)	Japan Public Health Center-Based Prospective *[Study [Study quality* a *by (Selection:3, Comparability:1, Outcome:2)]*
Johnson et al. 2011 [39]	Cohort	-	Canada	-	1994–2006	126	(185,100)	Using British Columbia Linked Health Database *[Study quality* a *by (Selection:4, Comparability:2, Outcome:2)]*
Hemminki et al. 2010 [32] b	Cohort	Swedish population	Sweden	>39	1964–2006	71 (2.9)	9,298	Standardized incidence rate, using hospital discharge register linking it to cancer register *[Study quality* a *by (Selection:3, Comparability:1, Outcome:2)]*
Atchison et al. 2010 [38]	Cohort	-	US	18∼100	1969–1996	1,053	(4,501,578)	Hospital discharge register linking it to cancer register *[Study quality* a *by (Selection:4, Comparability:1, Outcome:2)]*
Meinhold et al. 2009 [20] USRT study	Cohort	-	US	-	1982–2006	116 (Women)	(90,713)	US Radiologic Technologists Study for occupational irradiation exposure *[Study quality* a *by (Selection:3, Comparability:2, Outcome:2)]*
Lo et al. 2012 [41]	Cohort	Population in the same database	Taiwan	-	1996–2009	1,309	895,434	Taiwan National Health Research Institute (NHRI) database *[Study quality* a *by (Selection:3, Comparability:2, Outcome:2)]*
Kabat et al. 2012 [36]	Cohort	-	US		1993–2009	331 (Women)	159,009	Women's Health Initiative (WHI) study *[Study quality* a *by (Selection:3, Comparability:2, Outcome:2)]*
Stocks et al. 2009 d [34]	Cohort	-	Norway, Sweden, Austria	-	1972–2005	277	(2,738,701)	The Metabolic syndrome and Cancer project (Me-Can) *[Study quality* a *by (Selection:4, Comparability:1, Outcome:3)]*
Tulinius et al. 1997 [35]	Cohort	-	Iceland	-	1967–1995	46 (Women)	22,946	The Icelandic study of risk factors for cardiovascular disease (Reyjavik Study) *[Study quality* a *by (Selection:4, Comparability:2, Outcome:2)]*
Kitahara et al, 2012 [30]PLCO study c	Cohort		US	52–75	1993–2009	51	48,446	Prostate, Lung, Colorectal, and Ovarian Cancer Screening Trial *[Study quality* a *by (Selection:3, Comparability:0, Outcome:2)]*
Case-control studies								
Vecchia et al. 1994 [18]	Case-control	Hospital	Italy	<75	1983–1992	208	7,834	*[Study quality by (Selection:1, Comparability:1, Outcome:1)]*
Kuriki et al. 2007 [31]	Case-control	Hospital	Japan	>18	1988–2000	215	47,768	Data from the Hospital-Based Epidemiologic Research Program at Aichi Cancer Center, Japan (HERPACC) *[Study quality* a *by(Selection:2, Comparability:2, Outcome:2)]*
Duran et al. 2012 [33] e ^,^ f ^,^	Case-control	Hospital	Turkey	15–97	2003–2009	106^b^	2,224^b^	Data from single hospital of clinic of the Medical School at Baskent University *[Study quality* a *by (Selection:3, Comparability:1, Outcome:2)]*

NIH-AARP (National Institutes of Health-American Association of Retired Persons) study; USRT (United States Radiologic Technologists) study; PLCO (Prostate, lung, colorectal and Ovarian Cancer Screening Trial) study.

a Study quality was judged based on the Newcastle-Ottawa Scale (range, 1–9 stars).

b Standardized incidence ratio (SIR) per 1,000,000 within reference population.

c Prostate, lung, colorectal and Ovarian Cancer Screening Trial (PLCO) data.

d Participants who were classified into the highest quintile (quintile 5) were regarded as diabetic patients (including level for impaired fasting glucose metabolism).

e Participants with Impaired Fasting Glucose (IFG, 100≤FBS or OGTT<125) or Impaired Glucose Tolerance (IGT, 140≤OGTT≤199) by 2009 ADA criteria.

f Controls were benign thyroid diseases.

**Figure 2:**
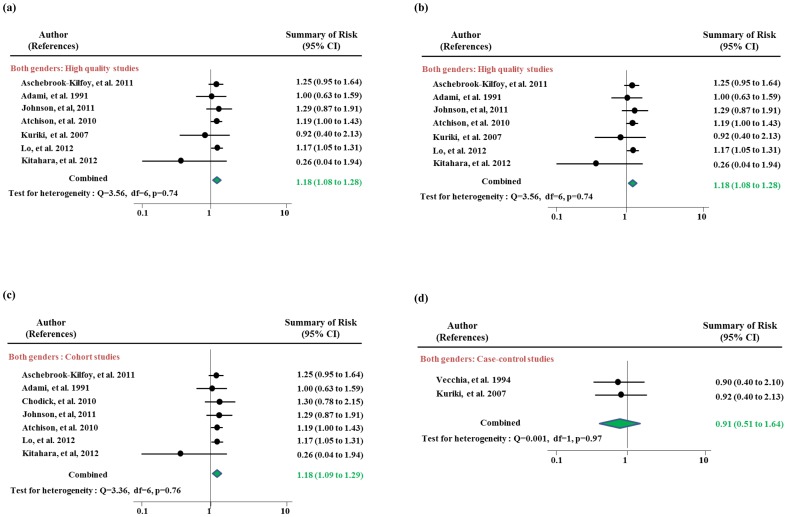
Meta-analysis of the association between diabetes mellitus and thyroid cancer in men and women: (a) all studies, (b) high quality studies (c) cohort studies and (d) case-control studies.

Table 2 and Figure 2 show risk estimates for DM-associated thyroid cancer risk in all studies and subgroups according to study design, geographic region, and study quality. People with type 2 DM were at an increased risk for thyroid cancer relative to non-diabetic people in all studies combined (RR = 1.34, 95% CI 1.11–1.63). However, there was heterogeneity across the studies (p-heterogeneity<0.0001). For the sensitivity analysis, we excluded the studies which reported risk estimates of SIR [14], [32] and had different definition of DM [33], [34]. When we excluded these studies, people in 9 studies remaining after sensitivity testing showed about a 20% increased risk of thyroid cancer associated with pre-existing DM (RR 1.18, 95% CI 1.08–1.28) (Figure 2-(a)). In the cohort studies, DM was associated with a greater increased risk for thyroid cancer (RR 1.18, 95% CI 1.09–1.09) without any heterogeneity (p for heterogeneity = 0.76) and no evidence for publication bias (p by Egger test = 0.39) (Figure 2-(b)). The risk estimate for case-control studies resulted in a relative risk of 0.91 (95% CI 0.51–1.64) which were estimated from the 2 studies. The results for studies from countries with a high incidence of thyroid cancer were similar to the results overall (RR 1.18, 95% CI 1.09–1.29). In low-rate geographic areas, the thyroid cancer risk associated with DM was no longer apparent. In high quality studies, type 2 DM was associated with a RR of thyroid cancer of 1.18 (95% CI 1.08–1.28) after sensitivity testing.

**Table 2:** Risk estimates for diabetes mellitus-associated thyroid cancer overall and within subgroups.

		N of studies	N of thyroid cancer cases	Summary RR (95% CI) a ^,^ c	p-heterogeneity
All studies		13	4,051	1.34 (1.11–1.63)	<0.001
	Sensitivity analysis b	9	3,566	1.18 (1.08–1.28)	0.84
Study design b	Cohort studies	7	3,143	1.18 (1.09–1.29)	0.76
	Case-control studies	2	423	0.91 (0.51–1.64)	0.97
Geographical area b	High incidence regions	7	3,446	1.18 (1.09–1.29)	0.76
	Low incidence regions	2	120	0.98 (0.66–1.47)	0.98
Study quality b	Score ≥6	7	3224	1.18 (1.08–1.28)	0.74
	Score <6	2	322	1.18 (0.76–1.81)	0.46

a All summary ORs/RRs (95% CIs) were calculated by the random-effect model.

b We excluded three studies using the risk estimates with SIRs ([14] and [32]) and the different definition of diabetes ([33]was included with IFG and IGT and [34] used quintile of glucose level).

c No publication bias by Egger and Begg test (p>0.05).

**Figure 3:**
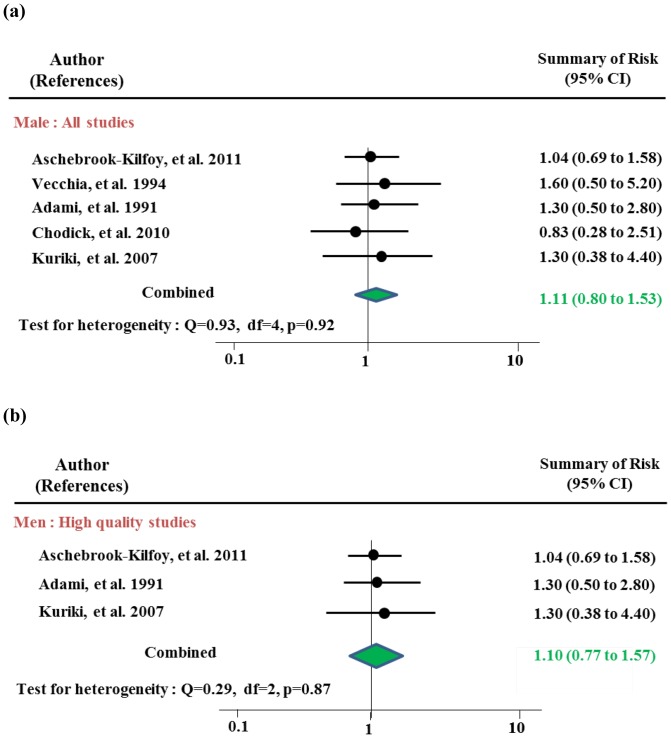
Meta-analysis of the association between diabetes mellitus and thyroid cancer in men: (a) all studies and (b) high quality studies.

**Figure 4:**
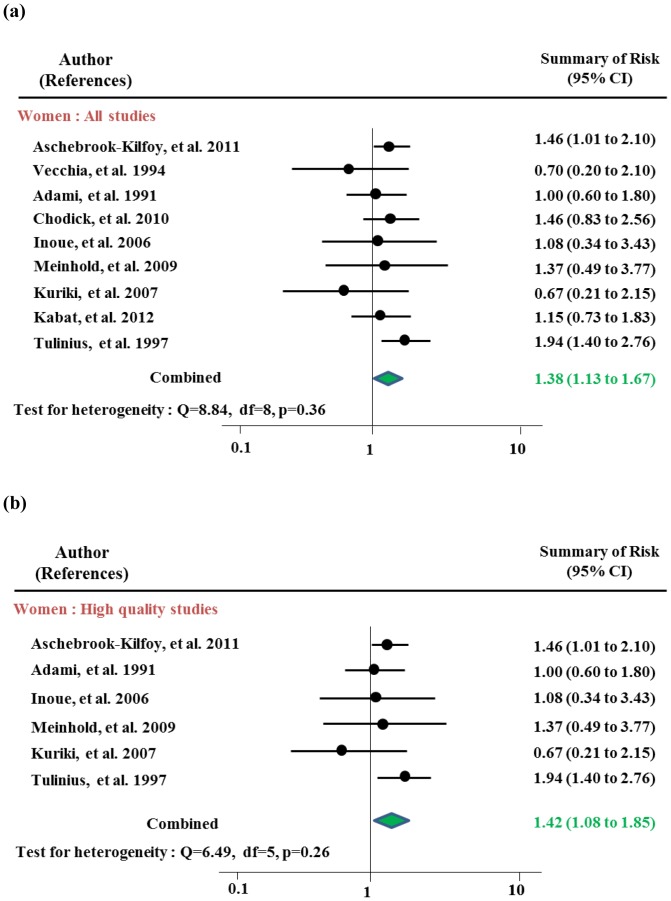
Meta-analysis of the association between diabetes mellitus and thyroid cancer in women: (a) all studies and (b) high quality studies.

Table 3 and Figure 3 and 4 present risk estimates stratified by gender. After sensitivity testing, women with type 2 DM had an increased risk of thyroid cancer of 1.38 (95% CI 1.13–1.67) overall and 1.42 (95% CI 1.08–1.85) among high quality studies, with risks among the cohort studies and in high incidence rates reached statistical significance Indication of publication was observed both in overall (p by Egger test = 0.01, respectively) which disappeared after excluding studies for sensitivity analysis. No publication bias was observed in sub-analyses. The risks among the cohort studies showed an increased risk of thyroid cancer with RR of 1.45 (95% CI 1.21–1.75). Rates of people in high incidence area were observed with RR of 1.32 (95% CI 1.04–1.68) without any heterogeneity. Men with DM were not at increased risk of thyroid cancer overall (RR 1.11, 95% CI 0.80–1.53) or in any of subgroup analysis strata after sensitivity analyses.

**Table 3:** Gender specific risk estimates for diabetes mellitus-associated thyroid cancer overall and within subgroups.

		N of studies	N of thyroid cancer cases	Summary RR (95% CI) a	p-heterogeneity
Women					
All studies		11	1,542	1.24 (0.98–1.58) c	0.11
	Sensitivity analysis b	9	1,244	1.38 (1.13–1.67)	0.36
Study design b	Cohort studies	7	929	1.45 (1.21–1.75)	0.44
	Case-control studies	2	315	0.69 (0.30–1.57)	0.96
Geographical area b	High incidence regions	6	1,055	1.50 (1.23–1.83)	0.40
	Low incidence regions	3	189	0.95 (0.60–1.50)	0.81
Study quality b	Score ≥6	6	687	1.42 (1.08–1.85)	0.26
	Score <6	3	557	1.20 (0.86–1.69)	0.52
Men					
All studies		7	506	1.15 (0.86–1.54)	0.49
	Sensitivity analysis b	5	219	1.11 (0.80–1.53)	0.92
Study design b	Cohort studies	3	111	1.06 (0.74–1.50)	0.81
	Case-control studies	2	108	1.45 (0.62–3.38)	0.81
Geographical area b	High incidence regions	3	148	1.06 (0.73–1.53)	0.71
	Low incidence regions	2	71	1.30 (0.64–2.63)	1.00
Study quality b	Score ≥6	3	123	1.10 (0.77–1.57)	0.87
	Score <6	2	96	1.13 (0.51–2.51)	0.42

a All summary ORs/RRs (95% CIs) were calculated by the random-effect model

b We excluded three studies using the risk estimates with SIRs ([14] and [32]) and the different definition of diabetes ([33] was included with IFG and IGT and [34] used quintile of glucose level)

c Publication bias by Egger and Begg test (p<0.05).

## Discussion

In this study, we investigated the association between type 2 DM and the incidence of thyroid cancer. The meta-analysis indicates that type 2 DM was associated with a statistically significant increase in thyroid cancer risk of approximately 20% of overall study populations, with a 30% increase among women, but not among men. This association was seen clearly in the cohort studies, in geographic areas in which there is a high incidence of thyroid cancer, and among high quality studies as measured by NOS scale.

A previous pooled analysis of five prospective studies [30] that included NIH-AARP, USRT, PLCO, AHS, and BCDDP reported no evidence of an association between a history of DM and thyroid cancer risk (HR = 1.08, 95% CI 0.83–1.40). The risk among women was higher risk (HR 1.19, 95% CI 0.84–1.69) but remained statistically non-significant. Subgroup analyses by smoking status, histologic type of thyroid cancer, educational attainment, and other factors all suggested no significant associations. The lack of association might be explained by the small number of cancer cases among the exposed group. In AHS and BCDDP, there were no thyroid cancer cases developing among persons with DM, and PLCO had only 1 thyroid cancer case in this group. In addition, all of these studies were conducted in the US, possibly limiting the heterogeneity of exposure and limiting statistical power. In our meta-analysis, we included studies conducted throughout the world, providing substantial population heterogeneity, and demonstrating that the risk associated with DM is most pronounced among women residing in areas which experience high rates of thyroid cancer relative to other geographic areas of the world.

Several biological mechanisms may account for this association. The first is via activation of insulin and the IGF pathway which share affinity with insulin and important to cell proliferation and apoptosis [42]. The chronically elevated circulating insulin levels associated with DM [43] may influence thyroid cancer risk mediated by insulin receptors overexpressed by cancer cells. IGF-1, a well-known pathway with an affinity for insulin, is also critical to cell proliferation and apoptosis and has been shown to be related to various types of cancer, such as breast and colon [44], [45]. A number of studies have also suggested that thyroid cancer risk is increased among persons with Metabolic Syndrome, which includes a glucose level in the diagnostic criteria [46]–[52]. Chronic metabolic disturbances, which are characteristics of type 2 DM and include aberrations in the insulin-like growth factor pathway, also affect steroid hormone metabolism suggesting that this pathway may also be involved [53]–[55].

A second possible mechanism involves long-term exposure to elevated thyroid-stimulating hormones (TSH). Concordant with the increase in anti-thyroid antibody level, primary hypothyroidism and the elevation of TSH, is 3 times more frequent in type 2 diabetics than in non-diabetics [56]. Even though the roles of TSH in thyroid carcinogenesis have not been established, there were several studies which reported the association of autonomous TSH regulation with reduction in thyroid cancer risk [57], or prediction of aggressive carcinoma of thyroid with higher TSH concentration [58]. Chronic high serum TSH concentration also predicted higher likelihood of differentiated thyroid cancer in that people whose TSH level were above the mean of population had higher risk of thyroid cancer compared to those with lower TSH level than the mean [59].

A third possible mechanism involves the impact of hyperglycemia on tumor cell growth and proliferation [60]. The possible mechanism is an increased oxidative stress [61] and as a metabolic factor, glucose can increase the production of reactive oxygen species, especially nitric oxide [62]. These seem to be much more complex because glucose metabolism is also influenced by sex hormones [63], [64]. The relationship between female reproductive hormones, glucose and thyroid cancer is still unclear. Recently, intracellular deiodinase, a regulating enzyme that controls expression of intracellular thyroid hormone levels, has been implicated as a potential carcinogenic mechanism in relation to diabetes and thyroid cancer [65], [66].

In our meta-analysis, the overall results showed heterogeneity across the studies. The heterogeneity problems disappeared after sensitivity analysis in all sub-group analyses and publication bias did not appear to be present. There were 2 studies which were excluded for using different criteria for DM in sensitivity analysis. In one study, blood glucose levels were inversely associated with thyroid cancer in women [34]. Since the quintile cut-points in this study were not provided, whether any of the quintiles were indicative of DM is not clear and may have resulted in the observed inverse association. Another one [33] is a case-control study with study population who conducted fine needle aspiration biopsy (FNAB) after confirmed thyroid mass. They reported the risk of malignant neoplasm compared with that of benign thyroid tumors as an exposure for glucose metabolism disorder, such as impaired glucose tolerance (IGT) and impaired fasting glucose (IFG) among those patients.

While the results for men was consistent, and there was no evidence for between study heterogeneity or publication bias, the lack of a statistically significant association between DM and thyroid cancer risk in this subgroup may simply be a result of a smaller number of cases and lack of information for prevalence of DM.

The possibility that the occurrence of thyroid cancer precedes the development of DM cannot be entirely excluded. The mean age for diagnosis of type 2 DM is similar to that of thyroid cancer around the age of 40 and since thyroid cancer is usually a slow-growing tumor and the diagnosis of DM can be delayed due to its often silent nature, the temporal sequence of which came first or the concurrent development of both cannot be ruled out even in cohort studies. The accurate time between incidence of DM and thyroid cancer is an important element in evaluating this relationship but is beyond the scope of this meta-analysis to examine it. Although, when provided, we used study results which excluded early incident cases.

Moreover, how long subjects were comorbid with DM and which drugs they used were not available in studies. Neither could we estimate whether they were under-controlled with DM or not. As hemoglobin A1C, one of the useful parameters that are usually examined for regular follow-up in clinics, was not available in the studies, we could not estimate any correlation of thyroid cancer risk with HbA1C. Duration of prevalent DM of participants, which might be related to the risk of thyroid cancer in the aspect of dose-response relationship, could not be taken into the analyses, either.

There are some additional potential limitations to this meta-analysis. Some studies which were included were based on patients' self-report. In addition, information on diabetes treatment was unknown, thus, controlled vs uncontrolled DM could not be distinguished. Some studies did not adjust for potentially confounding factors, such as obesity and age. In several studies when we estimated the RRs using the frequency data from the published tables, it was not available to adjust for potential confounding effects. Since DM is represented in most studies as a yes/no variable, we could not we could not characterize the shape of curve associated with different degrees of DM. Moreover, the overall results showed heterogeneity and publication bias was indicated across the studies among women. However, it was improved after excluding studies for sensitivity analysis. Finally, we were unable to conduct sub-group analysis for pathophysiologic types of thyroid cancer, thus, potentially attenuating risk estimates.

Nevertheless, this study had several strengths. We were able to conduct gender-specific analyses which suggested that the DM-thyroid cancer association may be more pronounced among women. If this is not simply a matter of statistical power, it may have implications for the mechanisms involved. In addition, we included studies that reported only glucose levels as the exposure of interest. We performed a sub-group analysis according to the type of risk estimates and found that the risk type did not influence on the direction or strength of thyroid cancer risk.

Our results indicate that DM may increase the risk of thyroid cancer in women. Thus, given the rapidly increasing risk of thyroid cancer worldwide, regular thyroid examination for type 2 DM patients may be worthwhile until these results can be further confirmed or clarified.

## Supporting Information

Table S1 **Risk estimates and their 95% confidence intervals in previous studies in relation to association between diabetes mellitus and thyroid cancer risk.**
(DOCX)

Table S2 **Judged study quality based on the Newcastle-Ottawa scale (range, 1-9 stars),**
(DOCX)

Checklist S1 **PRISMA Checklist 2009.**
(DOCX)
