# Supplementary material for: Diabetes Mellitus and Risk of Thyroid Cancer: A Meta-Analysis
Source: PLoS One. 2014 Jun 13;9(6):e98135. doi: 10.1371/journal.pone.0098135 (PMC4057085; doi:10.1371/journal.pone.0098135)
Supplement: Table S1 — Risk estimates and their 95% confidence intervals in previous studies in relation to association between diabetes mellitus and thyroid cancer risk. (DOCX) [file pone.0098135.s001.docx]

**Table S1. Risk estimates and their 95% confidence intervals in previous studies in relation to association between diabetes mellitus and thyroid cancer risk**

| Author [Reference] | Type 2 DM | Total | | | Women | | | Men | | | Adjusted variables |
| --- | --- | --- | --- | --- | --- | --- | --- | --- | --- | --- | --- |
|  |  | **N of cases** | **N of controls (cohort)** | **Risk estimates (95%CI)** | **N of cases** | **N of controls or cohorts**  **(person-years)** | **Risk estimates (95%CI)** | **N of cases** | **N of controls or cohorts (person-years)** | **Risk estimates (95%CI)** |  |
| Cohort Studies | |  |  |  |  |  |  |  |  |  |  |
| Aschebrook-Kilfoy et al. 2011 [[19](#_ENREF_19)] NIH-AARP | Never | 525 | 451,885 | 1.00 | 302 | 185,790 | 1.00 | 26 | 266,065 | 1.00 | Age, sex, smoking status, race, family history of cancer, BMI and education |
|  | Ever | 60 | 44,693 | 1.25 (0.95-1.64) | 34 | 14,766 | 1.46 (1.01-2.10) | 226 | 29,927 | 1.04 (0.69-1.58) |  |
| Wideroff et al. 1994 [[14](#_ENREF_14)] | Never | 31 | 109,581 | 1.00 | 21 | 55,010 | 1.00 | 10 | 54,571 | 1.00 | Sex, length of follow-up in years, and whether or not the hospital records mentioned obesity |
|  | Ever |  |  | 1.23 (0.84-1.81) **^a,b^** |  |  | 1.20 (0.70-1.80) **^b^** |  |  | 1.30 (0.60-2.30) **^b^** |  |
| Adami et al. 1991 [[17](#_ENREF_17)] | Never | 19 | 51,008 | 1.00 | 13 | 27,862  (143,618) | 1.00 | 6 | 23,146  (119,643) | 1.00 | Not reported |
|  | Ever |  |  | 1.00 (0.63-1.59) **^a^** |  |  | 1.00 (0.60-1.80) |  |  | 1.30 (0.50-2.80) |  |
| Chodick et al. 2010 [[40](#_ENREF_40)] | Never | 91 | 83,873 | 1.00 | 62 | 39,756 | 1.00 | 29 | 44,117 | 1.00 | Age, region, SES level, use of healthcare services a year prior to index date, BMI, and history of cardiovascular disease |
|  | Ever | 23 | 16,721 | 1.30 (0.78-2.15) **^a^** | 19 | 7,926 | 1.46 (0.83-2.56) | 4 | 8,795 | 0.83 (0.28-2.51) |  |
| Inoue et al. 2010 [[13](#_ENREF_13)] | Never |  |  |  | 100 | (543,313) | 1.00 | 0 | (458,524) | 1.00 | Age, study area (10 public health center areas), history of cerebrovascular disease, history of ischemic heart disease, smoking, ethanol intake, BMI, leisure-time physical activity |
|  | Ever |  |  |  | 3 | (16,247) | 1.08 (0.34-3.43) |  | (30,190) | N/A |  |
| Johnson et al. 2011 [[39](#_ENREF_39)] | Never | 52 | 185,100 | 1.00 |  |  |  |  |  |  | Age, sex, SES level, number of physician visit and year of diagnosis |
|  | Ever | 74 | 185,100 | 1.29 (0.87-1.91) |  |  |  |  |  |  |  |
| Hemminki et al. 2010 [[32](#_ENREF_32)] | Never | 71 | 9,298 | 1.00 |  |  |  |  |  |  | Expected number were calculated as age, sex, period, region and socioeconomic status-specific standard incidence rate |
|  | Ever |  |  | 2.24 (1.75-2.82) **^b^** |  |  |  |  |  |  |  |
| Atchison et al. 2010 [[38](#_ENREF_38)] | Never | 906 | (3,906,763) | 1.00 |  |  |  |  |  |  | Age, time, latency, race and the number of visits |
|  | Ever | 147 | (594,815) | 1.19 (1.00-1.43) |  |  |  |  |  |  |  |
| Meinhold et al. 2009 [[20](#_ENREF_20)] USRT study | Never |  |  |  | 112 | 69,506 | 1.00 | 16 | 21,207 | 1.00 | Birth year, smoking status, BMI, number of radiographs to the head and neck, occupational radiation dose and medical history of benign thyroid disease |
|  | Ever |  |  |  | 4 |  | 1.37 (0.49-3.77) | 0 |  | N/A |  |
| Lo et al. 2012 [[41](#_ENREF_41)] | Never | 620 | 895,434 | 1.00 |  |  |  |  |  |  | Age, sex, urbanization, hypertension, and hyperlipidemia |
|  | Ever | 689 | 895,434 | 1.17 (1.05-1.31) |  |  |  |  |  |  |  |
| Kabat et al. 2012 [[36](#_ENREF_36)] | Never |  |  |  | 312 | 149, | 1.00 |  |  |  | Crude RR recalculated from the data source |
|  | Ever |  |  |  | 19 | 9,400 | 1.06 (0.69-1.64) **^c^** |  |  |  |  |
| Stocks et al. 2009 [[34](#_ENREF_34)]^d^ | Never | 277 | (110,634) | 1.00 | 180 | (460,543) | 1.00 | 97 | (550,091) | 1.00 | Attained age as time scale, stratified by cohort, sex, and birth year, and adjusted for baseline age, BMI, and smoking status |
|  | Ever **^d^** |  |  | 1.33 (0.02-76.41) **^a^** |  |  | 0.18 (0.04-0.87) |  |  | 11.3 (1.29-98.3) |  |
| Tulinius et al. 1997 [[35](#_ENREF_35)] | Never |  |  |  | 46 | 11,580 | 1.00 | 37 | 11,366 | 1.00 | Recalculated using 99% confidence interval comparing the risk below the interval with above the interval, adjusted only for age |
|  | Ever |  |  |  |  |  | 1.94 (1.40-2.76) **^e^** |  |  | N/A |  |
| Kitahara et al., 2012 [[30](#_ENREF_30)] PLCO Study | Never | 50 | 45,053 | 1.00 |  |  |  |  |  |  | Adjusted for sex, education, race, marital status, smoking, BMI, and alcohol intake |
|  | Ever | 1 | 3,393 | 0.26 (0.04-1.94) |  |  |  |  |  |  |  |
|  | |  |  |  |  |  |  |  |  |  |  |
| Case-control studies | |  |  |  |  |  |  |  |  |  |  |
| Vecchia et al. 1994 [[18](#_ENREF_18)] | Never | 202 | 7,422 | 1.00 | 142 | 4,449- | 1.00 | 60 | 2,973 | 1.00 | Age and sex |
|  | Ever | 6 | 412 | 0.90 (0.40-2.10) | 3 | 252 | 0.70 (0.20-2.10) | 3 | 160 | 1.60 (0.50-5.20) |  |
| Kuriki et al. 2007 [[31](#_ENREF_31)] | Never | 209 | 45,844 | 1.00 | 167 | 32,622 | 1.00 | 42 | 13,222 | 1.00 | Age, BMI, drinking and smoking habits, regular physical exercise, bowel movement, history of cancer, family history of diabetes, dietary intake. |
|  | Ever | 6 | 943,302 | 0.92 (0.4-2.13) **^a^** | 3 | 777 | 0.67 (0.21-2.15) | 3 | 942,525 | 1.30 (0.38-4.40) |  |
| Duran et al. 2012 [[33](#_ENREF_33)] ^f^ | Never | 59 | 1,605 | 1.00 |  |  |  |  |  |  | Adjusted for age, sex and thyroid stimulating hormone level |
|  | Ever **^g^** | 47 | 629 | 2.85 (1.88-4.32) |  |  |  |  |  |  |  |

### NIH-AARP (National Institutes of Health-American Association of Retired Persons) study; USRT (United States Radiologic Technologists) study; PLCO (Prostate, lung, colorectal and Ovarian Cancer Screening Trial) study

### a. Meta-analysis results using the risk estimates in males and females

b. Standardized incidence ratio (SIR) per 1,000,000 within reference population

c. Recalculated crude relative risk (RR) by the number of non-diabetes/type 2 diabetes mellitus patients in the study

d. Participants who were classified into the highest quintile (quintile 5) were regarded as diabetic patients (including level for impaired fasting glucose metabolism)

e. The risk for participants whose glucose level were from 5.65 to upper limit of 99% confidence interval of exposed group (mean 5.83, SD 1.72, mmol/L) compared with those from lower limit of 99% CI to 5.65

f. Participants with Impaired Fasting Glucose (IFG, 100≤FBS or OGTT<125) or Impaired Glucose Tolerance (IGT, 140≤OGTT≤199) by 2009 ADA criteria

g. Controls were those with benign thyroid diseases
