# Supplementary material for: Diabetes Mellitus and Risk of Thyroid Cancer: A Meta-Analysis
Source: PLoS One. 2014 Jun 13;9(6):e98135. doi: 10.1371/journal.pone.0098135 (PMC4057085; doi:10.1371/journal.pone.0098135)
Supplement: Table S2 — Judged study quality based on the Newcastle-Ottawa scale (range, 1-9 stars), (DOCX) [file pone.0098135.s002.docx]

**Table S2. Judged study quality based on the Newcastle-Ottawa Scale (range, 1-9 stars)**

| Ref | Author | Selection | | | | | Comparability | | Outcome | |  | Total |
| --- | --- | --- | --- | --- | --- | --- | --- | --- | --- | --- | --- | --- |
|  |  | Case definition | | Representativeness of cases | Selection of Controls | Definition of controls | Control for main factor | Controls for additional factor | Ascertainment | Same  method | Non-response rate |  |
| Cohort studies | |  | |  |  |  |  |  |  |  |  |  |
| Aschebrook-Kilfoy, et al. 2011 [[19](#_ENREF_19)] NIH-AARP | | 1 | 1 | | 0 | 1 | 1 | - | 1 | 1 | 0 | 6 |
| Wideroff, et al.1994 [[14](#_ENREF_14)] | | 1 | 0 | | 1 | 1 | 0 | - | 1 | 1 | 0 | 5 |
| Adami, et al. 1991 [[17](#_ENREF_17)] | | 1 | 1 | | 1 | 1 | 1 | - | 1 | 1 | 0 | **7** |
| Chodick, et al. 2010 [[40](#_ENREF_40)] | | 1 | 1 | | 1 | 1 | 0 | - | 1 | 0 | 0 | 5 |
| Inoue, et al. 2010 [[13](#_ENREF_13)] | | 1 | 1 | | 0 | 1 | 1 | - | 1 | 1 | 0 | 6 |
| Johnson, et al. 2011 [[39](#_ENREF_39)] | | 1 | 1 | | 1 | 1 | 2 | - | 1 | 1 | 0 | 8 |
| Hemminki, et al. 2010 [[32](#_ENREF_32)] | | 1 | 0 | | 1 | 1 | 1 | - | 1 | 1 | 0 | 6 |
| Atchison, et al. 2010 [[38](#_ENREF_38)] | | 1 | 1 | | 1 | 1 | 1 | - | 1 | 1 | 0 | 7 |
| Meinhold et al. 2010 [[20](#_ENREF_20)] USRT study | | 1 | 1 | | 0 | 1 | 2 | - | 1 | 1 | 0 | 7 |
| Lo,et al. 2012 [[41](#_ENREF_41)] | | 1 | 1 | | 1 | 1 | 1 | - | 1 | 1 | 0 | 7 |
| Kabat, et al. 2012 [[36](#_ENREF_36)] | | 1 | 1 | | 0 | 1 | 0 | - | 0 | 1 | 0 | 5 |
| Stocks, et al. 2009 [[34](#_ENREF_34)] | | 1 | 1 | | 1 | 1 | 1 | - | 1 | 1 | 1 | 8 |
| Tulinius, et al. 1997 [[35](#_ENREF_35)] | | 1 | 1 | | 1 | 1 | 2 | - | 1 | 1 | 0 | 8 |
| Kitahara, et al. 2012 [[30](#_ENREF_30)] PLCO study | | 1 | 1 | | 0 | 1 | 0 |  | 1 | 1 | 0 | 6 |
| Case-control studies | | |  | |  |  |  |  |  |  |  |  |
| Vecchia, et al. 1994 [[18](#_ENREF_18)] | | 0 | | 0 | 0 | 1 | 1 | - | 0 | 1 | 0 | 3 |
| Kuriki, et al. 2007 [[31](#_ENREF_31)] | | 0 | | 1 | 0 | 1 | 2 | - | 0 | 1 | 1 | 6 |
| Duran, et al. 2012 [[33](#_ENREF_33)] | | 1 | | 1 | 0 | 1 | 1 | - | 1 | 1 | 0 | 6 |
